# Supplementary material for: Seasonal and Zonal Succession of Bacterial Communities in North Sea Salt Marsh Sediments
Source: Microorganisms. 2022 Apr 21;10(5):859. doi: 10.3390/microorganisms10050859 (PMC9146408; doi:10.3390/microorganisms10050859)
Supplement: Supplementary file 1 [file microorganisms-10-00859-s001.zip › microorganisms-1674921-supplementary.pdf]

microorganisms

MDPI

Supplements to:

Seasonal and Zonal Succession of Bacterial Communities in North Sea Salt Marsh Sediments

Dennis Alexander Tebbe [1], Simone Geihser [2], Bernd Wemheuer [2], Rolf Daniel [2], Hendrik Schäfer [3] and Bert Engelen [1,*]

[1] Institute for Chemistry and Biology of the Marine Environment, University of Oldenburg, Carl-von-Ossietzky-Straße 9-11, 26133 Oldenburg; bert.engelen@uol.de
[2] Institute of Microbiology and Genetics, Department of Genomic and Applied Microbiology, Georg-August Georg-August University of Göttingen, Göttingen, Germany; e-mail@e-mail.com
[3] School of Life Sciences, University of Warwick, Gibbet Hill Road, Coventry, CV4 7AL, United Kingdom; h.schaefer@warwick.ac.uk
* Correspondence: engelen@icbm.de

Table S1: GPS locations and heights in meters above mean sea level (MAMSL)

Location	Latitude	Longitude	MAMSL
Upp	53.76267804	7.722335574	1.4758
Low	53.76224433	7.722189404	1.4154
Pio1	53.76201613	7.722134075	1.3914
Pio2	53.76194715	7.722118738	1.2791
Pio3	53.76187794	7.722096806	1.2806
Pio4	53.76180422	7.72206909	1.1242
Pio5	53.76168163	7.721995092	0.8105
Edge	53.76157062	7.721945399	0.7241
Mud1	53.76144275	7.721770671	0.8299
Mud2	53.76104215	7.721726976	0.9456

Table S2: Permutational manova (PERMANOVA) results.

F.Model	R2	p.value	p.adjusted	sig	
DNA vs RNA	4.8409295	0.058436446	0.001	0.001	**
Upp vs Low	3.886039555	0.217266631	0.002	0.02	.
Upp vs Pio 1-5	4.658533799	0.091959507	0.001	0.01	*
Upp vs Edge	5.050657486	0.265117227	0.001	0.01	*
Upp vs Mud1-2	8.188077746	0.27123548	0.001	0.01	*
Low vs Pio1-5	2.846041803	0.058265556	0.001	0.01	*
Low vs Edge	5.422864662	0.279200044	0.001	0.01	*
Low vs Mud1-2	8.780242099	0.285255784	0.001	0.01	*
Pio1-5 vs Edge	4.084929479	0.081560053	0.001	0.01	*
Pio1-5 vs Mud1-2	9.204574874	0.145631466	0.001	0.01	*
Edge vs Mud1-2	1.191016685	0.051356812	0.267	1	
Sep14 vs Aug15	1.965413636	0.049177863	0.012	0.072	
Sep14 vs March15	3.244173196	0.078657734	0.001	0.006	*
Sep14 vs Oct15	1.963793542	0.049139318	0.011	0.066	
Aug15 vs March15	2.909782207	0.071126808	0.002	0.012	.
Aug15 vs Oct15	1.184706767	0.030233907	0.228	1	

Table S3: GPS locations and heights in meters above mean sea level (MAMSL)

Experiment Accession	Name in study	nucl type	Season	Library Name	Experiment Accession
SRX2847598	Pio3	DNA	Sep14	M2_Bacteria_DNA_T0_MiSeq	SRX2847598
SRX2847599	Pio5	DNA	Oct15	M4_Bacteria_DNA_T3_MiSeq	SRX2847599
SRX2847600	Pio5	RNA	Oct15	M4_Bacteria_RNA_T3_MiSeq	SRX2847600
SRX2847601	Low	DNA	Sep14	Low_Bacteria_DNA_T0_MiSeq	SRX2847601
SRX2847602	Pio2	DNA	Oct15	M1_Bacteria_DNA_T3_MiSeq	SRX2847602
SRX2847603	Pio4	DNA	Oct15	M3_Bacteria_DNA_T3_MiSeq	SRX2847603
SRX2847604	Pio4	RNA	Oct15	M3_Bacteria_RNA_T3_MiSeq	SRX2847604
SRX2847613	Mud2	RNA	March15	W2_Bacteria_RNA_T1_MiSeq	SRX2847613
SRX2847614	Mud2	DNA	March15	W2_Bacteria_DNA_T1_MiSeq	SRX2847614
SRX2847615	Mud1	RNA	March15	W1_Bacteria_RNA_T1_MiSeq	SRX2847615
SRX2847616	Mud1	DNA	March15	W1_Bacteria_DNA_T1_MiSeq	SRX2847616
SRX2847627	Low	RNA	Oct15	Low_Bacteria_RNA_T3_MiSeq	SRX2847627
SRX2847630	Pio3	DNA	Oct15	M2_Bacteria_DNA_T3_MiSeq	SRX2847630
SRX2847639	Low	DNA	Oct15	Low_Bacteria_DNA_T3_MiSeq	SRX2847639
SRX2847640	Pio2	RNA	Oct15	M1_Bacteria_RNA_T3_MiSeq	SRX2847640
SRX2847646	Pio3	RNA	Oct15	M2_Bacteria_RNA_T3_MiSeq	SRX2847646
SRX2847648	Pio2	DNA	March15	M1_Bacteria_DNA_T1_MiSeq	SRX2847648
SRX2847649	Pio2	RNA	March15	M1_Bacteria_RNA_T1_MiSeq	SRX2847649
SRX2847654	Pio3	DNA	March15	M2_Bacteria_DNA_T1_MiSeq	SRX2847654
SRX2847655	Pio3	RNA	March15	M2_Bacteria_RNA_T1_MiSeq	SRX2847655
SRX2847671	Pio1	DNA	Oct15	Pio_Bacteria_DNA_T3_MiSeq	SRX2847671
SRX2847672	Pio1	RNA	Oct15	Pio_Bacteria_RNA_T3_MiSeq	SRX2847672
SRX2847677	Pio5	RNA	March15	M4_Bacteria_RNA_T1_MiSeq	SRX2847677
SRX2847678	Pio5	DNA	March15	M4_Bacteria_DNA_T1_MiSeq	SRX2847678
SRX2847683	Pio4	RNA	March15	M3_Bacteria_RNA_T1_MiSeq	SRX2847683
SRX2847684	Pio4	DNA	March15	M3_Bacteria_DNA_T1_MiSeq	SRX2847684
SRX2847688	Mud2	RNA	Sep14	W2_Bacteria_RNA_T0_MiSeq	SRX2847688
SRX2847689	Mud2	DNA	Sep14	W2_Bacteria_DNA_T0_MiSeq	SRX2847689
SRX2847694	Mud1	RNA	Sep14	W1_Bacteria_RNA_T0_MiSeq	SRX2847694
SRX2847695	Mud1	DNA	Sep14	W1_Bacteria_DNA_T0_MiSeq	SRX2847695
SRX2847704	Pio4	RNA	Sep14	M3_Bacteria_RNA_T0_MiSeq	SRX2847704
SRX2847705	Pio4	DNA	Sep14	M3_Bacteria_DNA_T0_MiSeq	SRX2847705
SRX2847706	Upp	RNA	Sep14	Upp_Bacteria_RNA_T0_MiSeq	SRX2847706
SRX2847707	Pio5	RNA	Sep14	M4_Bacteria_RNA_T0_MiSeq	SRX2847707
SRX2847708	Pio5	DNA	Sep14	M4_Bacteria_DNA_T0_MiSeq	SRX2847708
SRX2847709	Upp	RNA	Oct15	Upp_Bacteria_RNA_T3_MiSeq	SRX2847709
SRX2847710	Upp	DNA	Oct15	Upp_Bacteria_DNA_T3_MiSeq	SRX2847710
SRX2847711	Low	DNA	March15	Low_Bacteria_DNA_T1_MiSeq	SRX2847711
SRX2847712	Low	RNA	March15	Low_Bacteria_RNA_T1_MiSeq	SRX2847712
SRX2847717	Upp	DNA	March15	Upp_Bacteria_DNA_T1_MiSeq	SRX2847717
SRX2847718	Upp	RNA	March15	Upp_Bacteria_RNA_T1_MiSeq	SRX2847718
SRX2847731	Edge	DNA	Sep14	M5_Bacteria_DNA_T0_MiSeq	SRX2847731
SRX2847732	Edge	RNA	Sep14	M5_Bacteria_RNA_T0_MiSeq	SRX2847732
SRX2847737	Low	RNA	Sep14	Low_Bacteria_RNA_T0_MiSeq	SRX2847737
SRX2847739	Pio3	RNA	Sep14	M2_Bacteria_RNA_T0_MiSeq	SRX2847739
SRX2847744	Pio2	DNA	Sep14	M1_Bacteria_DNA_T0_MiSeq	SRX2847744
SRX2847745	Pio2	RNA	Sep14	M1_Bacteria_RNA_T0_MiSeq	SRX2847745
SRX2847754	Pio1	RNA	March15	Pio_Bacteria_RNA_T1_MiSeq	SRX2847754
SRX2847755	Pio1	DNA	March15	Pio_Bacteria_DNA_T1_MiSeq	SRX2847755
SRX2847758	Mud2	DNA	Aug15	W2_Bacteria_DNA_T2_MiSeq	SRX2847758
SRX2847759	Mud2	RNA	Aug15	W2_Bacteria_RNA_T2_MiSeq	SRX2847759
SRX2847764	Mud1	DNA	Aug15	W1_Bacteria_DNA_T2_MiSeq	SRX2847764
SRX2847765	Mud1	RNA	Aug15	W1_Bacteria_RNA_T2_MiSeq	SRX2847765
SRX2847775	Pio1	RNA	Sep14	Pio_Bacteria_RNA_T0_MiSeq	SRX2847775
SRX2847776	Pio1	DNA	Sep14	Pio_Bacteria_DNA_T0_MiSeq	SRX2847776
SRX2847795	Edge	RNA	Aug15	M5_Bacteria_RNA_T2_MiSeq	SRX2847795
SRX2847796	Edge	DNA	Aug15	M5_Bacteria_DNA_T2_MiSeq	SRX2847796
SRX2847799	Low	DNA	Aug15	Low_Bacteria_DNA_T2_MiSeq	SRX2847799
SRX2847800	Low	RNA	Aug15	Low_Bacteria_RNA_T2_MiSeq	SRX2847800
SRX2847809	Upp	DNA	Aug15	Upp_Bacteria_DNA_T2_MiSeq	SRX2847809
SRX2847810	Upp	RNA	Aug15	Upp_Bacteria_RNA_T2_MiSeq	SRX2847810
SRX2847835	Upp	DNA	Sep14	Upp_Bacteria_DNA_T0_MiSeq	SRX2847835
SRX2847844	Pio1	RNA	Aug15	Pio_Bacteria_RNA_T2_MiSeq	SRX2847844
SRX2847845	Pio1	DNA	Aug15	Pio_Bacteria_DNA_T2_MiSeq	SRX2847845
SRX2847850	Mud1	DNA	Oct15	W1_Bacteria_DNA_T3_MiSeq	SRX2847850
SRX2847851	Mud1	RNA	Oct15	W1_Bacteria_RNA_T3_MiSeq	SRX2847851
SRX2847856	Mud2	DNA	Oct15	W2_Bacteria_DNA_T3_MiSeq	SRX2847856
SRX2847857	Mud2	RNA	Oct15	W2_Bacteria_RNA_T3_MiSeq	SRX2847857
SRX2847863	Pio4	DNA	Aug15	M3_Bacteria_DNA_T2_MiSeq	SRX2847863
SRX2847864	Pio4	RNA	Aug15	M3_Bacteria_RNA_T2_MiSeq	SRX2847864
SRX2847868	Pio3	RNA	Aug15	M2_Bacteria_RNA_T2_MiSeq	SRX2847868
SRX2847869	Pio3	DNA	Aug15	M2_Bacteria_DNA_T2_MiSeq	SRX2847869
SRX2847874	Pio2	RNA	Aug15	M1_Bacteria_RNA_T2_MiSeq	SRX2847874
SRX2847875	Pio2	DNA	Aug15	M1_Bacteria_DNA_T2_MiSeq	SRX2847875
SRX2847878	Edge	DNA	March15	M5_Bacteria_DNA_T1_MiSeq	SRX2847878
SRX2847879	Edge	RNA	March15	M5_Bacteria_RNA_T1_MiSeq	SRX2847879
SRX2847889	Pio5	DNA	Aug15	M4_Bacteria_DNA_T2_MiSeq	SRX2847889
SRX2847890	Edge	RNA	Oct15	M5_Bacteria_RNA_T3_MiSeq	SRX2847890
SRX2847891	Edge	DNA	Oct15	M5_Bacteria_DNA_T3_MiSeq	SRX2847891
SRX2847895	Pio5	RNA	Aug15	M4_Bacteria_RNA_T2_MiSeq	SRX2847895

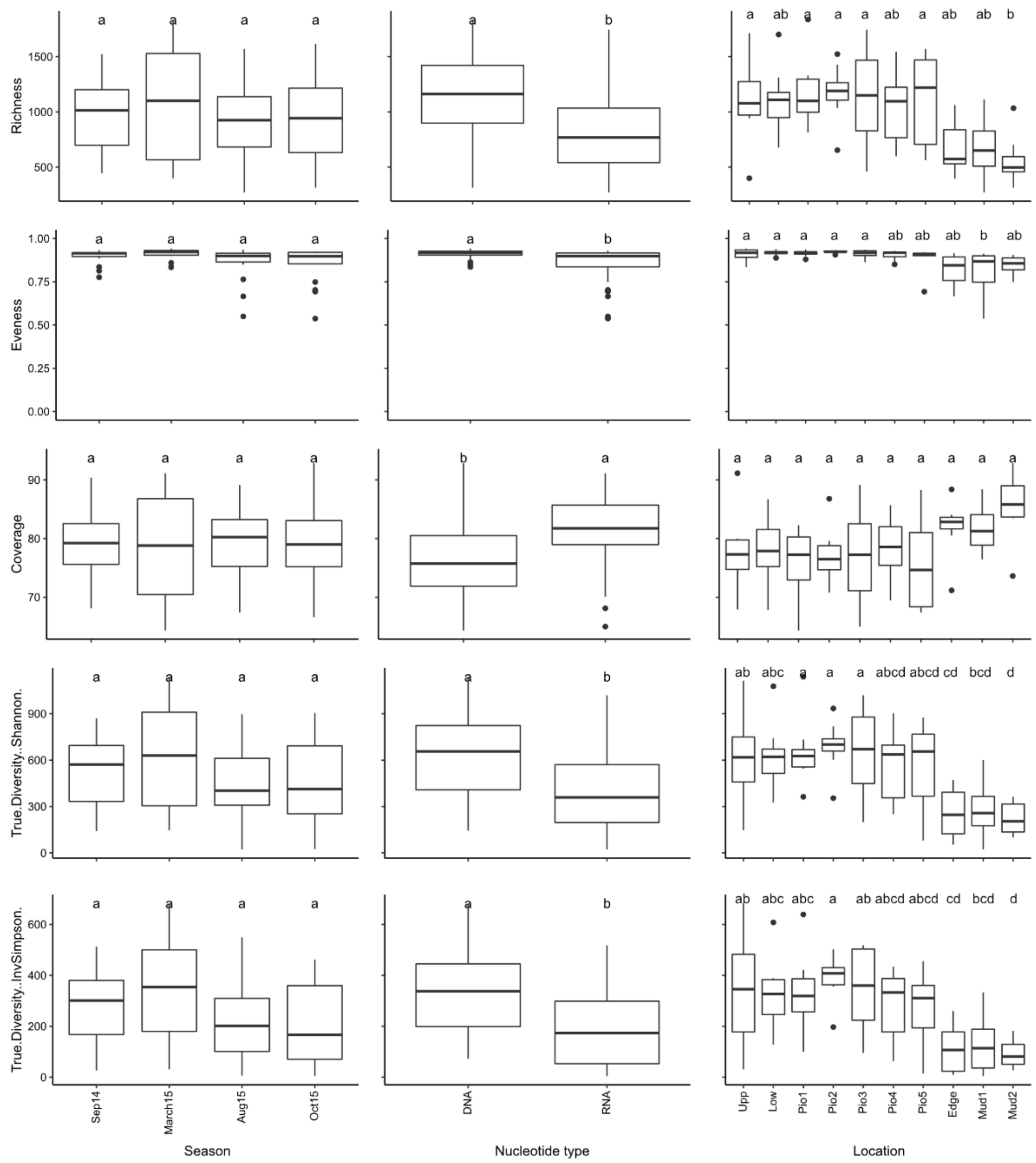

Figure S1: Alpha diversity measures. ASV were rarefied to 5909 and significance of differences in groups were tested with an analysis of variance (ANOVA) with $p <= 0.01$, followed by Tukey's HSD (honestly significant difference) test (alpha 0.05)

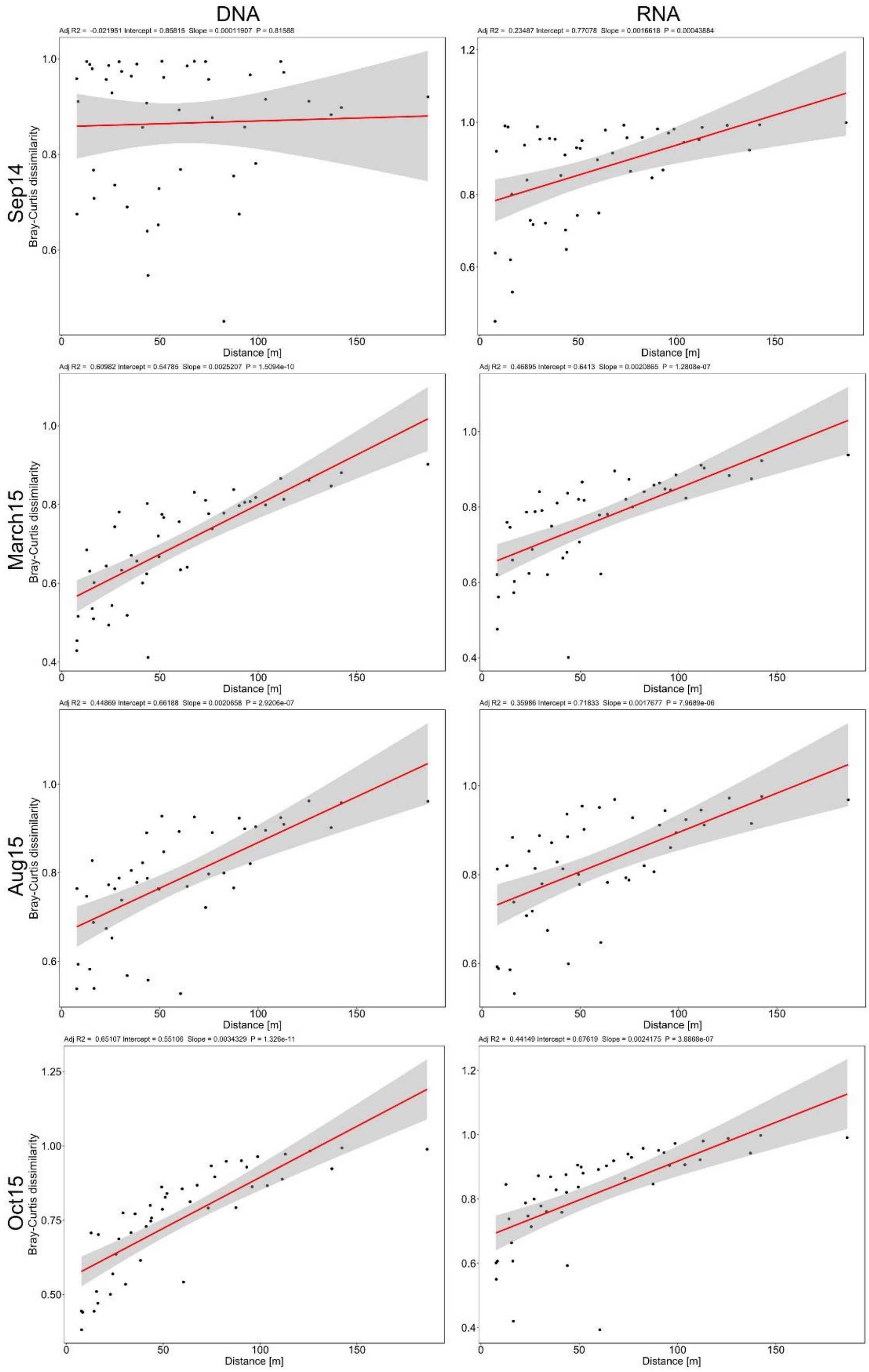

Figure S2: Distance-decay analysis. Pairwise Bray-Curtis dissimilarity over the geographic distance, independent of location. The red line represents a fitting linear model, with 95% confidence level interval as shaded area.

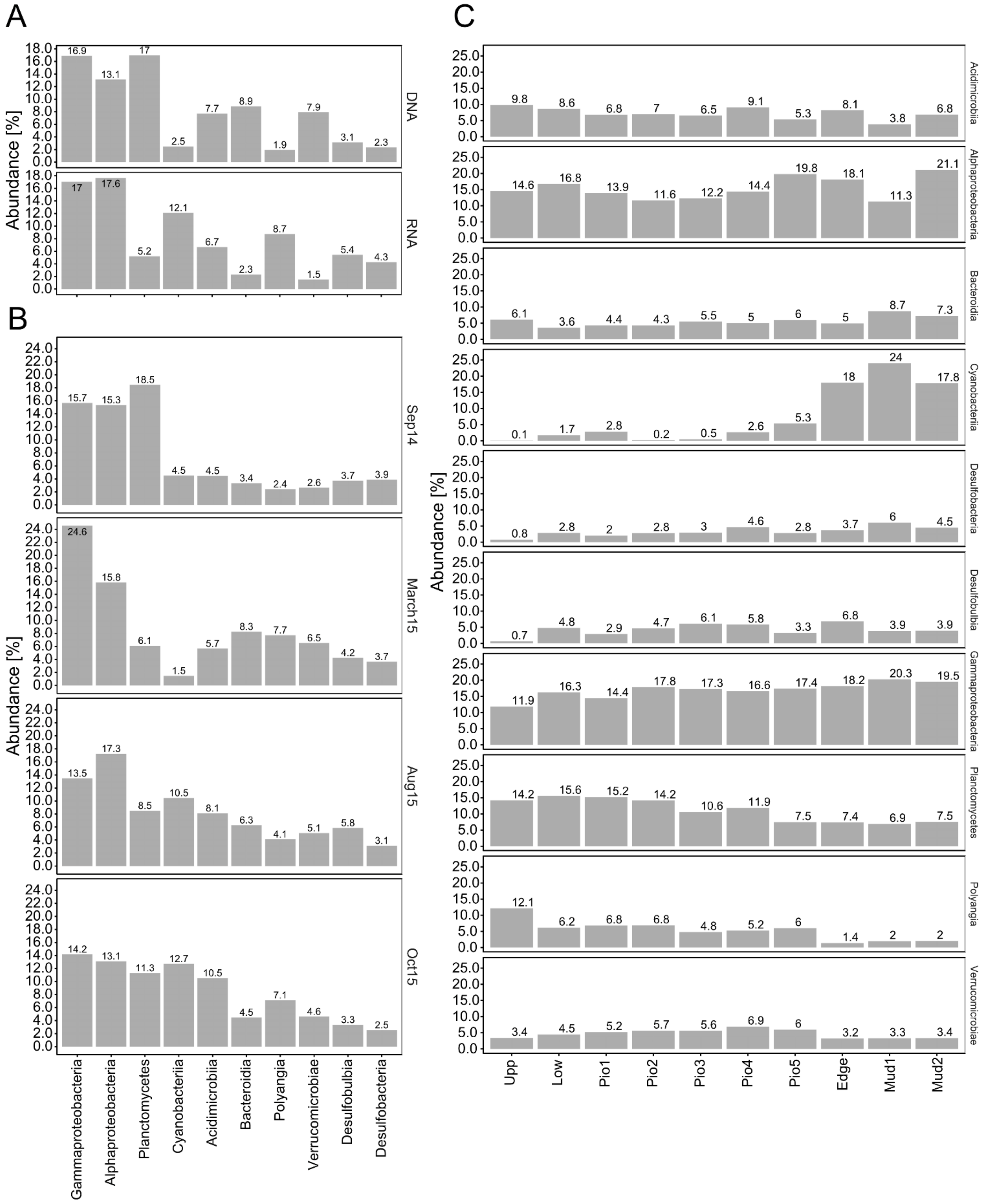

Figure S3: Top 10 most abundant taxa on Class level. (A) Means of all samples for DNA and RNA samples, (B) means of all samples per season, (C) means of all samples per sampling site.

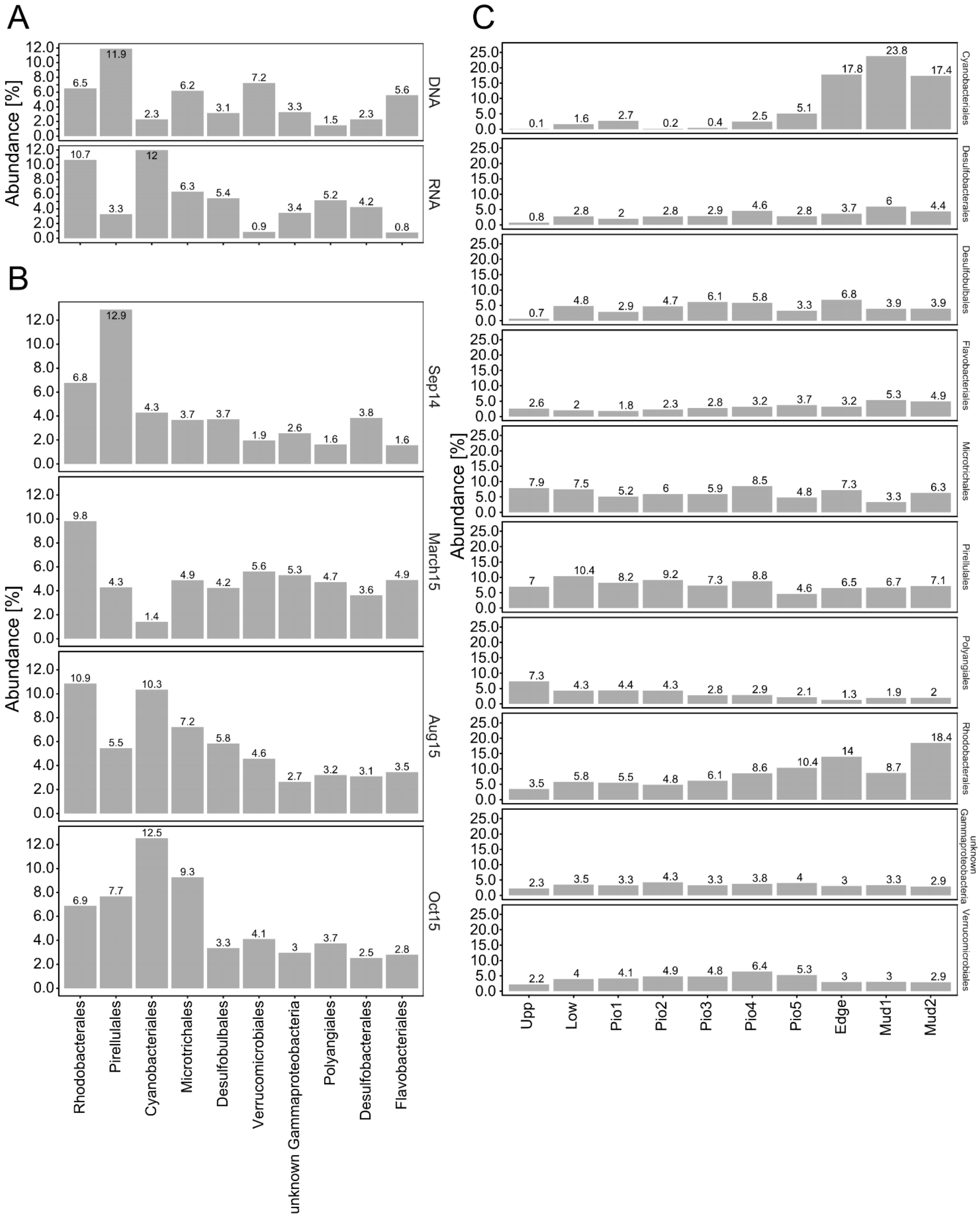

Figure S4: Top 10 most abundant taxa on Order level. (A) Means of all samples for DNA and RNA samples, (B) means of all samples per season, (C) means of all samples per sampling site.
